# Physiological and Behavioral Responses to Optogenetic Stimulation of PKD2L1^+^ Type III Taste Cells

**DOI:** 10.1523/ENEURO.0107-19.2019

**Published:** 2019-05-09

**Authors:** Courtney E. Wilson, Aurelie Vandenbeuch, Sue C. Kinnamon

**Affiliations:** Department of Otolaryngology, University of Colorado School of Medicine, Aurora, Colorado 80045

**Keywords:** optogenetics, taste, type III taste cells

## Abstract

Type III taste cells in mammalian taste buds are implicated in the detection and communication of sour and some salty stimuli, as well as carbonation and water. With this variety of proposed roles, it is unclear what information activated type III cells are communicating to the CNS. To better elucidate the role of type III cells in the taste bud, we use a type III cell-specific protein (polycystic kidney disease 2-like 1) to drive Cre-dependent expression of light-sensitive channelrhodopsin (Ai32) in mouse type III taste cells. Activation of these cells with light produces a taste nerve response in both the chorda tympani and glossopharyngeal nerves, and elicits a slight but significant aversion in two-bottle preference tests in both male and female mice. Unlike previous reports (Zocchi et al., 2017), our mice did not react to blue light stimulation with sustained drinking responses. These data suggest that type III cells are capable of communicating the presence of aversive stimuli in the oral cavity, which is in line with their responsiveness to sour and high concentrations of salt stimuli.

## Significance Statement

The sour-sensing type III cells of the mammalian taste bud are historically difficult to study *in vivo*, as sour stimulation of the tongue causes intracellular acidification in all epithelial cells, not just type III cells. The present study circumvents this issue by using Cre-dependent expression of channelrhodopsin in type III cells, so that they can be activated by blue light, rather than acid (sour). Our data suggest that type III cells communicate an aversive signal to the CNS.

## Introduction

Taste buds are the sensory end organs of the gustatory system, allowing organisms to discern nutritious energy sources from potentially dangerous ones. In most mammals, these clusters of sensory cells detect at least five basic taste modalities: bitter, sweet, umami, sour, and salty. Some sensory cells in the taste bud process discreet modalities; each type II cell in the taste bud transduces only bitter, sweet, or umami stimuli, and communicates this information to afferent nerve fibers via nonvesicular ATP release (Finger et al., 2005; Taruno et al., 2013; Romanov et al., 2018). Type I cells are thought to play primarily a support role in the taste bud similar to glial cells, with enzymes for uptake and degradation of transmitters (Lawton et al., 2000; Bartel et al., 2006; Dvoryanchikov et al., 2009). Here we focus on type III cells, which are implicated in a variety of functions in the taste bud.

Type III cells are elongated, spindle-shaped cells featuring synaptic connections with afferent nerve fibers (Murray et al., 1969). Since their identification as distinct ultrastructural components of the taste bud, these cells have been associated with several physiologic properties. Type III cells express the polycystic kidney disease 2-like 1 (PKD2L1) ion channel (Kataoka et al., 2008), and, although the function of this protein remains unclear, it has been used as a marker of type III cells and the *Pkd2l1* gene has been used as a Cre driver to manipulate gene expression. Genetic deletion of PKD2L1-expressing taste cells eliminates chorda tympani nerve responses to sour (acidic) taste stimuli (Huang et al., 2006), but type III cells have also been implicated in responses to carbonation (Chandrashekar et al., 2009) and high concentrations of salt (Oka et al., 2013; Lewandowski et al., 2016), all of which are considered aversive modalities. However, a recent study (Zocchi et al., 2017) entailing expression of channelrhodopsin-2 (ChR2) challenged the negative valence of type III cells, suggesting instead that type III cells primarily mediate water detection and drive drinking behavior. The mouse used in their study was produced from a BAC-transgene containing the *Pkd2l1* locus, which was then used to drive ChR2 in type III cells. On blue light stimulation of the tongue, mice exhibited continuous licking, even in the absence of water in the sipper tube. Zocchi et al. (2017) suggested that the averseness of acids may result not from the activation of type III cells, but from additional mechanisms of acid detection in the tongue, such as trigeminal afferents.

We have developed a similar mouse to manipulate gene expression in type III cells, also using the *Pkd2l1* gene as a Cre driver. However, we made our mice by knockin of an IRES Cre recombinase construct directly following the *Pkd2l1* stop codon (Ye et al., 2016). This mouse was characterized and used to knock down the potassium channel K_IR_2.1, validating its role in sour taste transduction (Ye et al., 2016). In the present study, we have used this mouse to re-examine the role of type III cells in taste behavior. We crossed our *Pkd2l1*-Cre mice to floxed ChR2 mice, validated the expression of ChR2 immunohistochemically, and characterized the nerve response resulting from light stimulation of the tongue. To assess behavior, we used two-bottle preference tests and brief-access Lickometer tests during optogenetic stimulation of the tongue. Our results indicate that our *Pkd2l1*-Cre, ChR2 mice avoid blue light stimuli in two-bottle preference tests and, contrary to the previous study, show no sustained licking responses to light stimulation. These data suggest that selective activation of PKD2L1^+^ type III cells elicits primarily a negative valence, as would be expected for a sour stimulus.

## Materials and Methods

### Mice

All mice were housed at the University of Colorado Anschutz Medical Campus on a 12 h light/dark cycle and had continual access to standard chow. The Animal Care and Use Committee at the University of Colorado School of Medicine approved all procedures. To drive the expression of the light-sensitive ChR2 in PKD2L1^+^ type III cells, we crossed a Cre-dependent ChR2 mouse line (stock #012569, The Jackson Laboratory) to a Pkd2l1-Cre mouse created in house (Ye et al., 2016). This knock-in mouse features an IRES Cre-recombinase construct directly following the *Pkd2l1* coding sequence. For behavioral experiments, littermate controls lacked one or both of the necessary alleles for ChR2 expression in PKD2L1^+^ type III cells.

### Perfusion/fixation

To fix and obtain taste tissues, mice were anesthetized with sodium pentobarbital via intraperitoneal injection at 50 mg/kg and transcardially perfused with 4% paraformaldehyde (PFA; catalog #158127, Sigma-Aldrich). Tongues were extracted and immersed in 4% PFA for 1.5–5 h. Tongues were then transferred to a 20% sucrose solution overnight at 4**°**C before being mounted in optimal cutting temperature compound (Thermo Fisher Scientific) and cut into 12–16 µm slices via cryostat. Tissue was collected onto charged slides (Tanner Scientific) in a 1:10 series and stored at −20**°**C.

### Immunohistochemistry

Before antibody staining, slides were washed in 0.1 m PBS (monobasic sodium phosphate, catalog #S-5011, Sigma-Aldrich; dibasic sodium phosphate, catalog #S-0876, Sigma-Aldrich; sodium chloride, catalog #S-7653, Sigma-Aldrich) three times for 10 min on a gentle shaker. A blocking solution of 2% normal donkey serum in blocking buffer (0.1 m PBS + 0.3% Triton X-100, catalog #22686, USB; 1% bovine serum albumin, catalog #A-7906, Sigma-Aldrich) was applied at room temperature, in darkness, for 1 h. Slides were incubated with one of the listed primary antisera (Table 1) in blocking buffer. For control slides, primary antisera were excluded. All slides were then washed in 0.1 m PBS three times for 10 min. Secondary antibodies were applied to each slide in blocking buffer for 3 h, in darkness, at room temperature (Table 2). The addition of DRAQ5 (catalog #ab108410, Abcam) at 1:5000 and/or DAPI (catalog #03571, Thermo Fisher Scientific) at 1:10,000 allowed for visualization of cell nuclei and identification of taste buds. Slides were subsequently washed in 0.1 m PBS and 0.05 m PB before applying coverslips (Fluoromount-G, catalog #0100-01, Southern Biotech; catalog #48393 251, VWR).

**Table 1.** List of primary antisera

Target protein	Host	Dilution	Manufacturer	Catalog #	RRID	Lot
GFP	Chicken	1:2000	Aves	GFP-1020	AB_10000240	0511FP12
PKD2L1	Rabbit	1:500	Hiroaki Matsunami Laboratory, Duke University Medical Center	PKD2L1	AB_2661860	N/A
SNAP25	Goat	1:1000	GeneTex	GTX89577	AB_10724125	821604337
P2X_3_	Rabbit	1:200	Alomone Labs	APR-016	AB_2313760	APR016AN0802
5-HT	Rabbit	1:2500	Immunostar	20080	AB_572263	1431001
PLCβ2	Rabbit	1:200	Santa Cruz Biotechnology	Sc-206	AB_632197	A1204

**Table 2.** List of secondary antisera

Target species	Host	Dilution	Manufacturer	Catalog #	RRID	Wavelength
Chicken	Donkey	1:400	Jackson ImmunoResearch	703-545-155	AB_2340375	488
Rabbit	Donkey	1:400	Molecular Probes	A10042	AB_11180183	568
Goat	Donkey	1:400	Molecular Probes	A21447	AB_141844	647

### Imaging and cell counting

Tissues were imaged using a 40× oil immersion numerical aperture 1.25 lens on a Leica S5 confocal microscope, with Leica LAS AF software version 2.7.3.9723. Cells were counted by first converting each channel into a binary image via a modified Otsu method in ImageJ (version 1.49, NIH public domain), combining channels to form a composite image, and counting cells using the ImageJ plug-in cell counter. Profiles were considered to be cells of interest if, in either marker channel, the profile had both: (1) an apparent nucleus; and (2) an elongate apical process. Profiles were considered positive for a marker if any part of the profile contained fluorescence. Venn Diagram Plotter (Kyle Littlefield, DOE, 2004) was used to create to-scale Venn diagrams, and Photoshop, Illustrator, and InDesign (CS6, Adobe Systems) were used to compose photographs and figures.

### Nerve recording

Mice were anesthetized with urethane at 2 g/kg (catalog #U2500, Sigma-Aldrich) and stabilized spatially with a custom head holder. A tracheotomy was performed to facilitate breathing during tongue stimulation. The chorda tympani nerve was approached ventrally, cut near the tympanic bulla, and placed on a platinum-iridium wire electrode. For glossopharyngeal recordings, the glossopharyngeal nerve was accessed ventrally, near the trachea. A reference electrode was inserted into nearby tissue. Nerve responses were elicited by applying tastants to the anterior or posterior tongue using a pump (Mini-pump, variable flow, Thermo Fisher Scientific). Tastants included NH_4_Cl 100 mm (A661, Thermo Fisher Scientific), citric acid 10 mm (catalog #C0759, Sigma-Aldrich), and sucrose 500 mm (catalog #S5016, Sigma-Aldrich). Photic stimuli were delivered via a 600 nm patch cord made by the Optogenetics and Neural Engineering Core at the University of Colorado School of Medicine [Optogenetics and Neural Engineering (ONE) Core] during constant water flow with fiber-coupled LED light sources in blue and amber wavelengths (product #M470F3 and #M595F2, Thorlabs), driven by T-Cube LED Drivers (Compact T-Cube LED Driver, Thorlabs) and a 7 MHz DDS Function Generator (product #4007B, BK Precision). Stimuli were applied for 30 s, followed by 60 s of water rinse. Nerve responses were amplified (P511, Grass Instruments), integrated over a time constant of 0.5 s, and recorded using Acknowledge software (Biopac). In most cases, responses were quantified by measuring the mean of the integrated response over 30 s from the onset of the stimulus, such that both the transient and tonic portions of the nerve response were included. For experiments involving a varied duty cycle, the length of stimulation was altered to normalize the total time of light applied to the tongue. When the duty cycle is at 50%, 5 Hz light pulses result in 100-ms-long light pulses exposing the tongue to a total of 15 s of light during the 30 s stimulation. Decreasing the duty cycle reduces the total time of light exposure, so the stimulus period was increased to compensate for this, and so on. In select experiments, purinergic receptor blocker AF353 (Afferent Pharmaceuticals) was perfused over the mouse tongue for 10 min at a concentration of 1 mm between full tastant sets to block nerve responses, and in conjunction with tastants for the remainder of the experiment.

### Behavior: two-bottle preference tests

Mice were water deprived for 24 h before the test period. In two-bottle preference tests, two sipper bottles, each containing water or a tastant solution, were presented to each mouse in their home cage for 15 min to alleviate the effect of side preference; each preference score was calculated from 2 test days, for which the tastant placement in the cage was reversed. For behavioral experiments involving light, optogenetic fibers were adapted (with the assistance of the ONE Core at the University of Colorado Anschutz Campus) to fit inside sipper bottles so that light was emitted from within the sipper spout. Pulsed light (5 Hz, 7 mW, 50% duty cycle) was delivered in the same manner as for nerve recordings. All statistics and graphs were performed/generated with Prism 7 (GraphPad Software), and formatted with Illustrator and Indesign (CS6, Adobe Systems).

### Behavior: lickometer studies

As in two-bottle preference tests, mice were water deprived for 24 h before the test period. A laser replaced the previously described LED system (SLOC BL473T3-050FC), allowing for a higher power output (35 mW), similar to that used previously (Zocchi et al., 2017). As in two-bottle preference tests, the optic fiber was adapted for a sipper tube that fit into a Davis Rig (DiLog Instruments), where the mice were given access to either an empty bottle without light, an empty bottle with constant blue light, or a bottle with water but without light for 10 min, and their licks were measured by InstaCal software. All statistics and graphs were performed/generated with Prism 7 (GraphPad Software) and formatted using Illustrator and Indesign (CS6, Adobe Systems).

### Experimental design and statistical analysis

**Figure 1.**
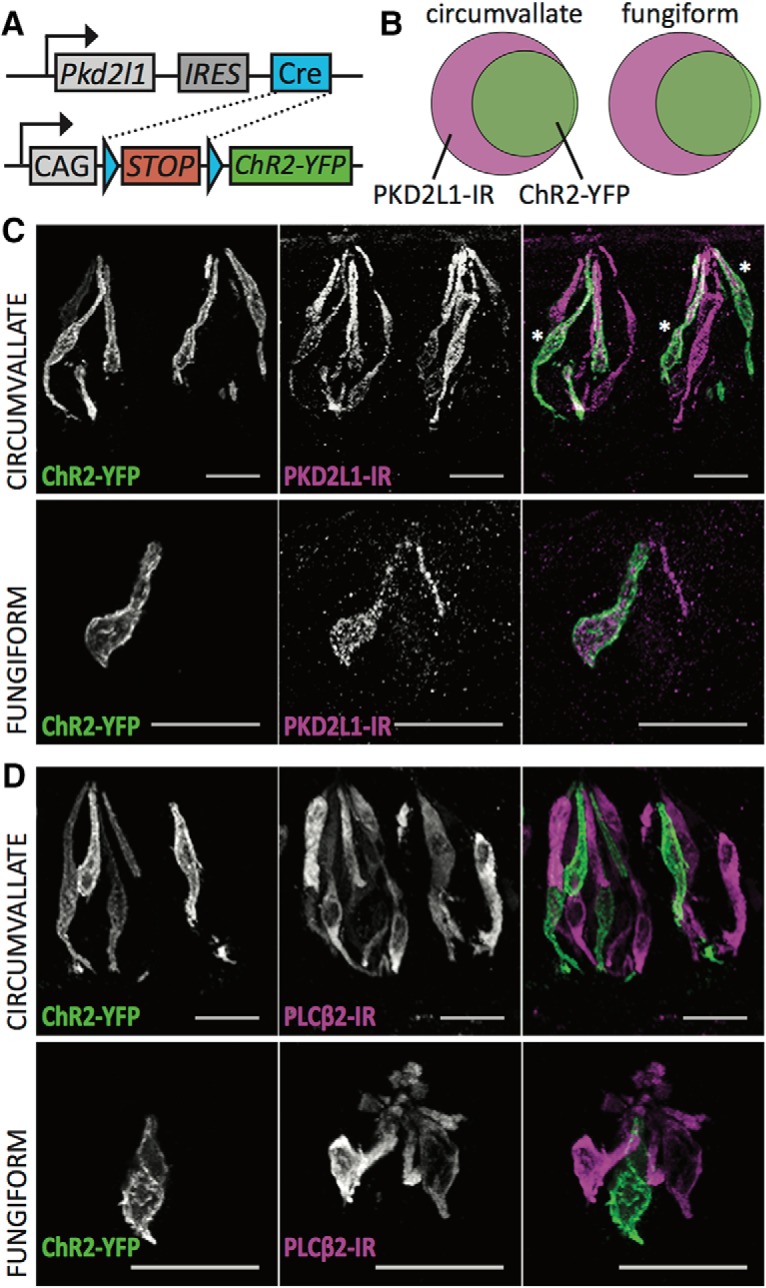
*Pkd2l1*-Cre drives ChR2-YFP specifically in most PKD2L1-immunoreactive cells. ***A***, Genetic construction of the *Pkd2l1*-Cre, ChR2-YFP mouse. ***B***, Venn diagrams illustrating the coincidence of PKD2L1 immunoreactivity (IR; magenta) and ChR2-YFP fluorescence (green) in circumvallate (left) and fungiform (right) taste tissues. Cell counts are as follows: for circumvallate, PKD2L1-IR only = 175 cells, ChR2-YFP only = 4 cells, both = 202 cells; for fungiform, PKD2L1-IR only = 14 cells, ChR2-YFP only = 1 cell, both = 14 cells. ***C***, Confocal *z*-stack images showing ChR2-YFP fluorescence in green and PKD2L1-IR in magenta in both the circumvallate (top) and fungiform (bottom) taste tissues. Because PKD2L1-IR tends to localize in the apical region of the taste cell, some cell bodies appear more green than magenta, but nonetheless exhibit PKD2L1-IR. Three such cells are marked with asterisks in the merged image. ***D***, Confocal *z*-stack images showing the separation of ChR2-YFP fluorescence in green and type II cell marker PLCβ2-IR in magenta in both the circumvallate (top) and fungiform (bottom) taste tissues. Scale bars, 20 µm.

**Figure 2.**
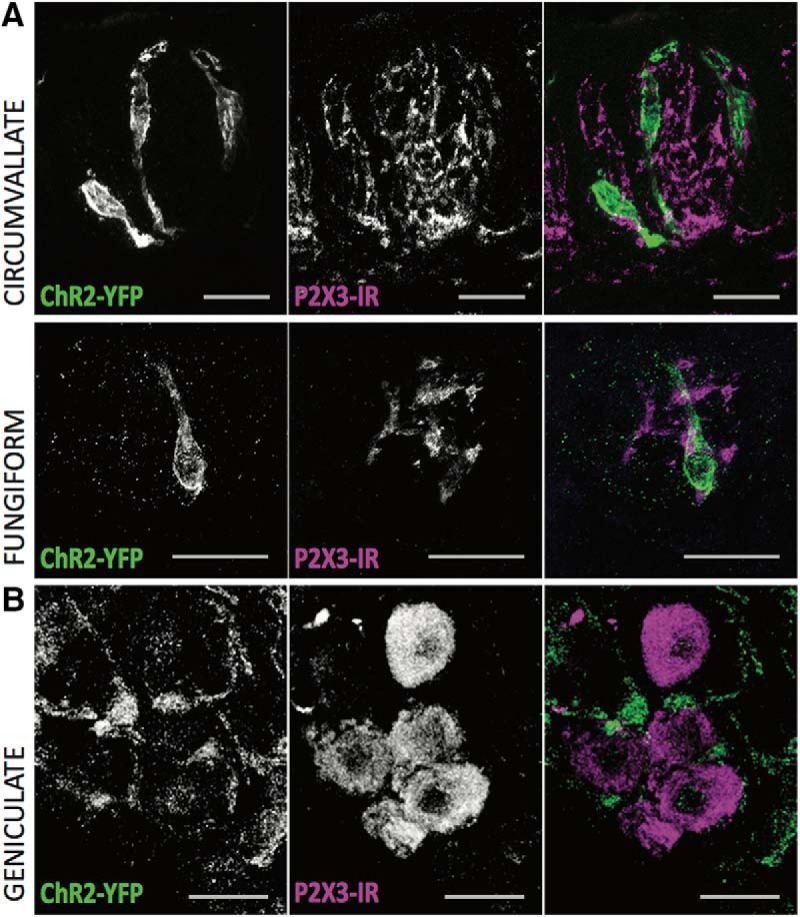
ChR2-YFP is not expressed in afferent taste nerve fibers or the ganglion cell bodies of the geniculate. ***A***, Confocal *z*-stack images of circumvallate and fungiform taste buds showing ChR2-YFP fluorescence in green and taste nerve marker P2X_3_-immunoreactivy (IR) in magenta. ***B***, Confocal *z*-stack images of geniculate ganglion cells showing ChR2-YFP fluorescence in green and taste ganglion cell marker P2X_3_-IR in magenta. Although green fluorescence appears in the ganglion, it seems to be restricted to satellite cells and is not present in neuronal cell bodies. Scale bars, 20 µm.

**Figure 3.**
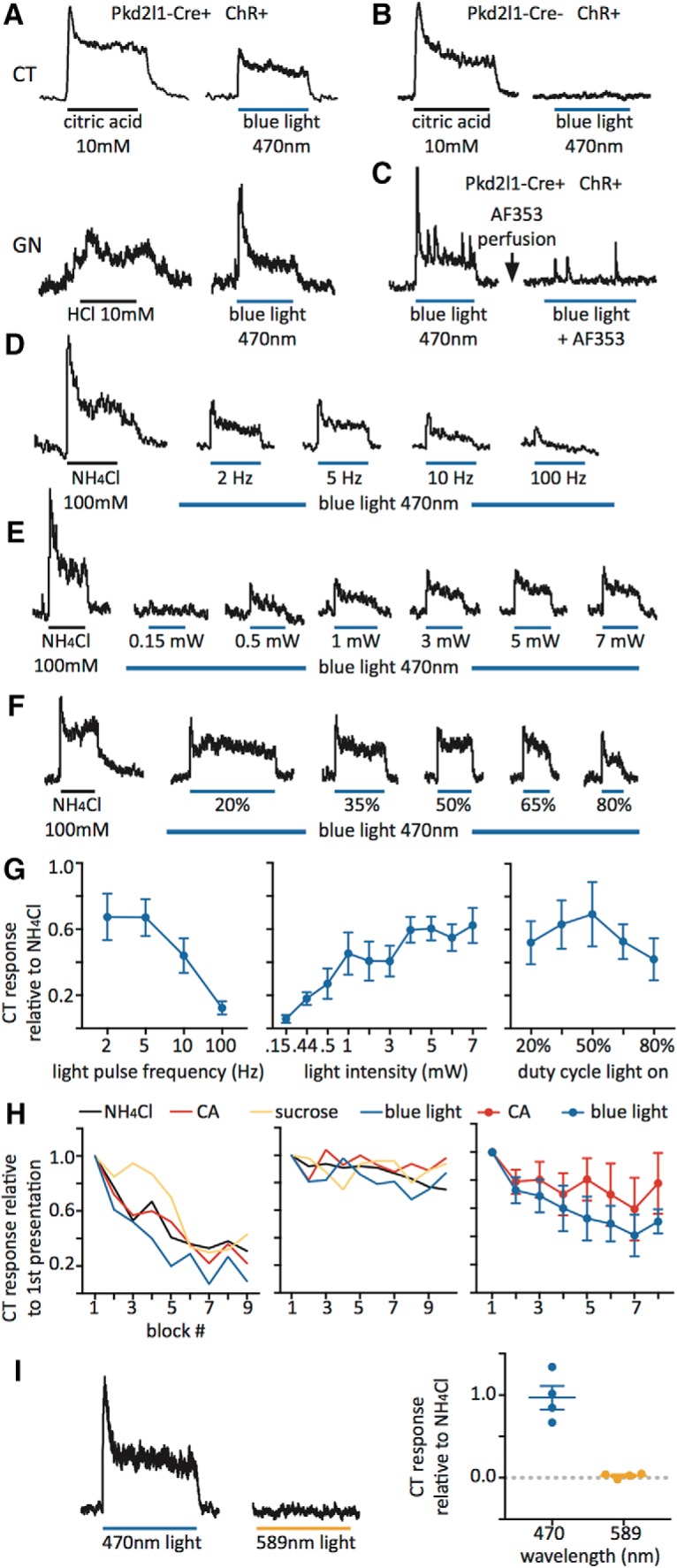
Optogenetic stimulation of the tongue in *Pkd2l1*-Cre, ChR2 mice elicits a robust, tastant-like nerve response. ***A***, Chorda tympani (CT) and glossopharyngeal (GN) nerve responses to acidic stimuli and a 470 nm light pulse stimulus in *Pkd2l1*-Cre, ChR2 mice. ***B***, CT nerve responses to citric acid and blue light in a Cre-negative control mouse. ***C***, CT nerve responses to blue light in a *Pkd2l1*-Cre, ChR2 mouse before and after the application of purinergic receptor blocker AF353 to the tongue. ***D***, CT nerve responses to a control tastant stimulus (NH_4_Cl) and blue light pulses at increasing frequencies (power at 7 mW, duty cycle at 50%). ***E***, CT nerve responses to NH_4_Cl and blue light pulses at increasing light power (frequency at 5 Hz, duty cycle at 50%). ***F***, CT nerve responses to NH_4_Cl and blue light pulses at increasing light-on duty cycle percentages (frequency at 5 Hz, power at 7 mW). ***G***, All quantified data in frequency (*n* = 3), power (*n* = 4), and duty cycle experiments (*n* = 3). ***H***, Two example experiments (left and middle) showing the consistency of the blue light response, compared with repeated blocks of 100 mm NH_4_Cl (black), 10 mm citric acid (CA; red), 500 mm sucrose (yellow), and blue light at 5 Hz pulses, 7 mW power, and 50% duty cycle (blue). All presentations normalized to the first presentation of that stimulus (e.g., CA responses normalized to first presentation of CA, light responses normalized to first presentation of light). Right graph shows all data (*n* = 6) comparing the consistency of blue light responses (blue) to CA responses (red). Blue light responses were not different from CA responses over presentation blocks (*F*_(7,70)_ = 0.254, *p* = 0.9692, two-way ANOVA). ***I***, CT nerve responses to blue (470 nm) and amber (589 nm) light. Graph on right shows all data (*n* = 4) of varied wavelength stimulation. Stimulus bars denote 30 s of stimulation, except for those in ***F***, which are adjusted to normalize total time of light exposure to the tongue. All error bars denote the SEM.

**Figure 4.**
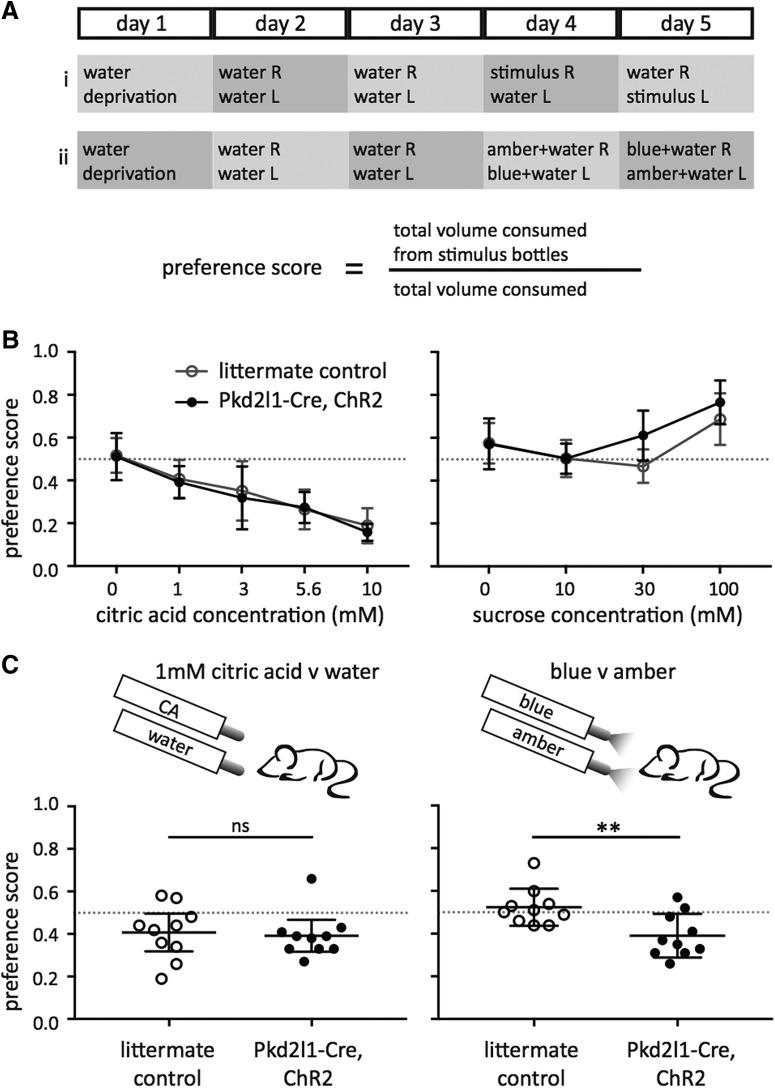
*Pkd2l1*-Cre, ChR2 mice avoid blue light activation compared with amber control light presentation. ***A***, Behavioral experiment sequence for (1) a typical taste stimuli two-bottle preference test, and (2) a blue v amber light two-bottle preference test. Preference score calculation at bottom. ***B***, Taste preference curves for varying concentrations of citric acid (left) and sucrose (right) in littermate controls (gray, open), and *Pkd2l1*-Cre, ChR2 mice (black, filled). Scores >0.5 indicate a preference for the taste stimulus, while those <0.5 indicate an avoidance. ***C***, Preference scores for two-bottle preference tests for 1 mm citric acid (left) and blue light (v amber, right) with littermate controls (open circles), and *Pkd2l1*-Cre, ChR2 mice (filled circles). Both *Pkd2l1*-Cre, ChR2 mice and controls slightly avoided 1 mm citric acid, but were not significantly different from each other (*t* = 0.31, **p = 0.7601, unpaired *t* test). *Pkd2l1*-Cre, ChR2 mice avoided blue when compared to amber light, while controls did not (*t* = 3.137, *p* = 0.0057, unpaired *t* test). All error bars denote 95% confidence intervals.

**Figure 5.**
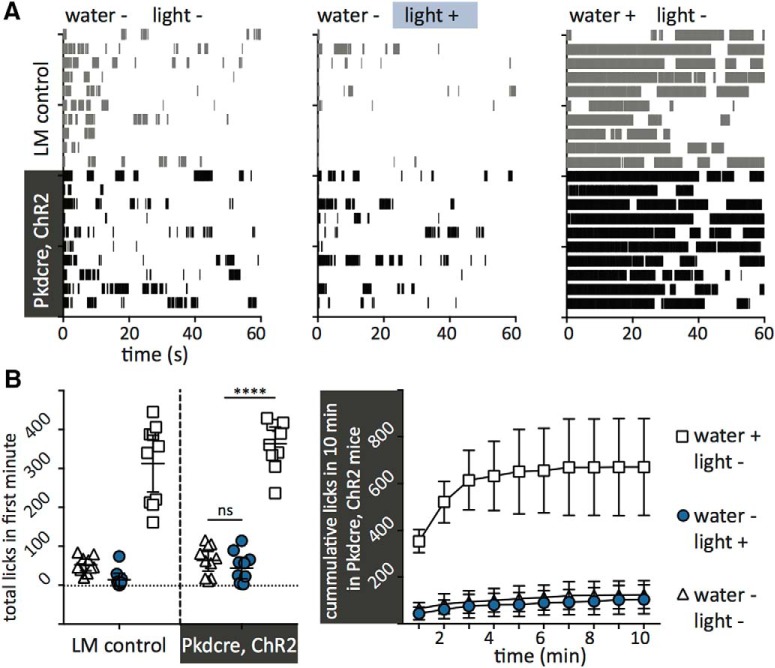
*Pkd2l1*-Cre, ChR2 mice do not increase licking behavior in response to light in the absence of water. ***A***, Lick patterns during the first minute of behavioral experiments with 10 littermate control (gray) and 10 *Pkd2l1*-Cre, ChR2 (black) mice featuring a water spout without water or light (left), without water but with blue light (center), or with water and without light (right). ***B***, Left, Total licks in the first minute of the experiments in ***A***. For *Pkd2l1*-Cre, ChR2 mice (black), total licks did not differ between the water− light− condition and the water− light+ condition (*t* = 1.135, *p* = 0.2712, unpaired *t* test). Lick totals in both of these conditions were significantly different from the lick count for the water+ light− condition (*t* = 13 and *t* = 14, respectively; ****p < 0.0001 for each, unpaired *t* test). Right, Cumulative lick total over 10 min for *Pkd2l1*-Cre, ChR2 mice under each condition. All error bars denote 95% confidence intervals.

For immunohistochemical studies designed to evaluate the efficiency of Cre-driven expression in PKD2L1^+^ cells, taste tissues from seven mice (three female, four male) were imaged and analyzed. Tissues from two mice (one female, one male) were stained and imaged to determine whether *Pkd2l1-*Cre drives misexpression in type II taste cells (Fig. 1). Tissues from two additional mice (one female, one male) were stained and imaged to determine whether *Pkd2l1-*Cre drives misexpression in taste nerve fibers or cell bodies (Fig. 2). Per mouse, 4 sections of circumvallate tissue and ∼12 sections of fungiform tissue were examined, and any taste buds therein were imaged. Nerve recording experiments featured a mix of female and male mice, totaling 24 mice, presented in Figure 3. Light frequency data include three mice, all female. Light power data include four mice (two female, two male). Light duty cycle data include three mice, all female. Data collected to assess the reliability of the light response include six mice (four female, two male). To compare the reliability of the light response to traditional liquid tastant responses, a two-way ANOVA was performed between light responses and pooled liquid tastant responses (Prism 7, GraphPad Software). To determine whether amber light could also elicit a nerve response, recordings of nerve responses to blue and amber light were performed and included four mice (three female, one male). For behavioral experiments presented in Figures 4 and 5, 20 age- and sex-matched mice were used to collect data (10 control mice, 10 *Pkd2l1-*Cre, ChR2 mice; 14 total females, 6 total males). In Figure 4, preference scores of control mice were compared with those of *Pkd2l1-*Cre, ChR2 mice by unpaired *t* tests. In Figure 5, lick counts in the first minute of the experiments were compared between conditions by unpaired *t* tests. In all experiments, no differences due to the sex of the animals were observed.

## Results

### Channelrhodopsin expression in the Pkd2l1-Cre, ChR2 mouse

The Pkd2l1-Cre mouse successfully drives ChR2 in approximately half of PKD2L1^+^ type III cells. In circumvallate taste tissues of five mice, ∼54% of PKD2L1-immunoreactive cells were also positive for ChR2-yellow fluorescent protein (YFP) fluorescence. In fungiform tissues of the same mice, 50% of PKD2L1-immunoreactive cells displayed ChR2-YFP fluorescence (Fig. 1*A–C*). Importantly, ChR2-YFP fluorescence does not appear in cells immunoreactive for type II cell marker PLCβ2 (Fig. 1*D*). ChR2-YFP fluorescence is likewise absent from P2X_3_-immunoreactive nerve fibers innervating circumvallate and fungiform taste buds (Fig. 2*A*). Examination of the geniculate ganglion, which contains cell bodies of taste neurons, reveals some ChR2-YFP fluorescent signal. This signal, however, is restricted to satellite cells that do not overlap with the P2X_3_-immunoreactive, rounded cell bodies characteristic of taste sensory neurons (Fig. 2*B*). Optogenetic stimulation of ChR2 cells, therefore, is unlikely to directly stimulate any non-type III cells or gustatory nerve fibers.

### Optogenetic stimulation of PKD2L1^+^ cells elicits taste nerve responses

Pulsed blue light directed toward either the anterior or posterior tongue of an anesthetized mouse elicits a nerve response in the chorda tympani or glossopharyngeal nerve similar to those elicited by tastant solutions. These responses are only elicited in mice containing both the *Pkd2l1*-Cre and Cre-dependent ChR2-YFP alleles, and are blocked by purinergic receptor blocker AF353, which eliminates responses to all tastant solutions (Vandenbeuch et al., 2015; Fig. 3*A–C*). After testing various parameters of light presentation, we determined that blue light pulses at 5 Hz frequency, 4*–*7 mW of power, and a 50% duty cycle (when the light is on for half of the cycle period) elicited a maximal chorda tympani response. For experiments testing frequency, power and duty cycle were kept at 7 mW and 50%, respectively. For power dose–response curves, frequency and duty cycle were set at 5 Hz and 50%, respectively. For duty cycle experiments, frequency and power were set at 5 Hz and 7 mW (Fig. 3*D–G*). To test for the consistency of this response, we conducted several experiments repeating the same block of tastants: NH_4_Cl 100 mm, citric acid 10 mm, sucrose 500 mm, and blue light pulses at 5 Hz, 7 mW, and 50% duty cycle (each presentation of four stimuli constitutes a “block”). While the consistency of tastant responses for separate experiments/animals varied (Fig. 3*H*, left and middle) the chorda tympani response to blue light stimulation remained approximately as reliable as other tastants for the course of an experiment. Citric acid responses over several blocks did not differ from averaged light responses (Fig. 3*H*, right). Amber light (589 nm) does not elicit a chorda tympani nerve response (Fig. 3*I*).

### Behavioral responses to optogenetic activation of PKD2L1^+^ cells

To determine whether *Pkd2l1*-Cre, ChR2 mice prefer or avoid optogenetic stimulation of type III cells, we devised an experimental paradigm to test taste preference for light, rather than traditional tastant solution preferences (Fig. 4*A*). Light was always presented in conjunction with water in the sipper tube. Both *Pkd2l1*-Cre, ChR2 mice, and littermate controls avoided increasing concentrations of citric acid, and preferred increasing concentrations of sucrose (Fig. 4*B*). In two-bottle preference tests including light, amber light was included as a control to avoid visual behavioral preference, as amber light is within the mouse visual range (Jacobs 1993; Neitz and Neitz 2001) but does not elicit a nerve response. *Pkd2l1*-Cre and ChR2 mice, but not littermate controls, avoided blue light in favor of amber light. The level of avoidance is comparable to that of low concentrations of citric acid.

Experiments performed with a similar mouse by Zocchi et al. (2017) showed an intriguing result—blue light stimulation of the tongue during behavioral experiments elicited a strong licking behavior, even in the absence of water. As these results seem contrary to our own, we sought to better replicate those experiments. We measured single-lick events of *Pkd2l1*-Cre, ChR2, and littermate control mice in a Davis rig, where the mice were presented with sipper bottles without water or blue light, without water and with light, or with water and without blue light. A laser light source replaced the LED light source to better mimic the power output and geometry of light application described in Zocchi et al. (2017). While *Pkd2l1*-Cre, ChR2 mice licked more in response to light than littermate controls, the total number of licks in the first minute was not different between the with- and without-light conditions, and both were significantly different from the with-water, without-light condition (Fig. 5). Over the full 10 min trial, *Pkd2l1*-Cre, ChR2 mice presented with water were eventually satiated and discontinued drinking, while mice stopped licking the spout soon after the start of the trial when water was not present (Fig. 5*B*, right).

## Discussion

In the present study we show that optogenetic stimulation of PKD2L1^+^ type III taste cells elicits a nerve response that resembles responses to tastants and is somewhat aversive to the awake, behaving mouse. Our *Pkd2l1*-Cre mouse drives ChR2-YFP expression in approximately half of PKD2L1-immunoreactive cells, and does not drive the expression in type II cells or taste nerve fibers or cell bodies. Thus, we are confident that this activation is specific to type III cells. Photic stimulation of the tongue elicits taste-like responses in the chorda tympani and glossopharyngeal nerves. This blue light-induced nerve response is blocked by the purinergic receptor blocker AF353 (Vandenbeuch et al., 2015), similar to nerve responses to traditional tastant solutions. The response to blue light is maximal at a 5 Hz pulse with a 50% duty cycle at light powers of ≥4 mW. Perhaps owing to the moderate expression efficiency in PKD2L1-immunoreactive cells (Fig. 1), the nerve response elicited by blue light is smaller than that elicited by 10 mm citric acid, which is near the EC_50_ value for citric acid (Arai et al., 2010). In two-bottle preference tests, *Pkd2l1*-Cre, ChR2 mice slightly avoid blue light, while control littermates are indifferent. This avoidance behavior is akin to the avoidance of low concentrations of citric acid—both are slightly, but consistently, aversive to the group of mice we tested. These data contribute to the body of evidence that identifies type III taste cells as part of a generally aversive signaling pathway (Huang et al., 2006; Oka et al., 2013).

That type III cells communicate the presence of an aversive stimulus is consistent with previous data indicating their involvement in the transduction of sour and high-salt stimuli. Sour is thought to be an indication of the freshness of a food source; rotting foods are rife with acid-producing microorganisms (DeSimone et al., 2001). Salt homeostasis is crucial for survival, so detecting and avoiding sources of damaging excess salt is likewise important. It follows that the activation of cells that respond to these qualities would induce avoidance behaviors. The degree to which our *Pkd2l1*-Cre mice avoided blue light in comparison with amber light was quite modest—a feature that may be due to experimental limitations or might indicate the particular role of the anterior tongue in taste-related behavior. Our *Pkd2l1*-Cre driver drove ChR2 expression in most, but not all PKD2L1^+^ type III cells, which are less populous in the anterior tongue (Yoshida et al., 2009). Since the circumvallate taste buds of the posterior tongue are more difficult to stimulate with light, it is unlikely that our stimulation paradigm successfully activated as many posterior type III cells as anteriorly situated ones. Alternatively, the modest avoidance we see may indicate the role of anterior tongue in behavioral responses to tastants. Several lines of evidence suggest that the anterior tongue may be important for discriminating and identifying specific tastants, while the posterior tongue acts more as a binary gatekeeper (Travers et al., 1987; Frank, 1991; Spector and Grill 1992; Hellekant et al., 1997; St John and Spector 1998; Tomchik et al., 2007; Yoshida et al., 2009). Anterior tongue, therefore, may not communicate aversion so much as stimulus identity.

Our behavioral data differ considerably from recent findings, which suggest that PKD2L1^+^ cells are water detectors as well as sour detectors (Zocchi et al., 2017). Recent studies observe that type III taste cells are more heterogeneous than previously assumed (Wilson et al., 2017; Lossow et al., 2017). This underlying molecular diversity within the type III cell population is unlikely to cause such divergent results as seen between this work and that of Zocchi et al. (2017), because both studies targeted the same genetic subpopulation. However, while both studies use *Pkd2l1* to drive ChR2 expression, the tactics are slightly different; Zocchi et al. (2017) used a BAC transgenic mouse, which drives Cre expression from the *Pkd2l1* promoter region, while we used a mouse featuring an IRES-Cre knock-in construct directly following the *Pkd2l1* gene. These separate approaches may give rise to differences in expression levels and/or patterns, both of which might affect behavioral responses to light stimulation of the oral cavity. Our mouse, for example, drives ChR2 expression in only about half of the PKD2L1-immunoreactive cells. Perhaps a subtype of PKD2L1^+^ type III cells that was not activated in our experiments is responsible for water detection, and the behaviors observed by Zocchi et al. (2017) are the result of activation of this distinct population. ChR2 expression level differences between the two mouse models might also affect the level of activation of type III cells. Increased amounts of ChR2 in a single cell might increase the baseline electrical excitability on account of some constitutive activity of the channels. Differential type III cell excitability may, in turn, alter signaling to afferent nerve fibers. The synapse between type III cells and afferent nerve fibers has thus far been treated as a sort of “on/off” switch, but the nervous system often uses more nuanced methods of information transfer. Neurons involved in lower-frequency sound localization in gerbils, for example, code information not by maximal firing rate, but by a gradated slope of firing rates (for review, see Ashida and Carr, 2011), and retinal bipolar cells generally function as integrators of currents without firing action potentials (for review, see Nelson and Connaughton, 2007). Perhaps coding at the type III cell–nerve synapse depends in part on the degree of depolarization, allowing for a more complicated transmission paradigm that may differ between our mouse models. Ultimately, more experimentation is necessary to tease out the causes for our differing results.

The data presented here demonstrate that direct activation of PKD2L1^+^ type III cells by light is sufficient to elicit responses in nerves innervating the anterior and posterior tongue, as well as aversive behavioral responses. Continued experimentation with this mouse model is necessary to determine the perceived taste quality of this optogenetic stimulation and to resolve the apparent conflicts with published data.

## Synthesis

Reviewing Editor: Anna Menini, SISSA (International School of Advances Studies) Decisions are customarily a result of the Reviewing Editor and the peer reviewers coming together and discussing their recommendations until a consensus is reached. When revisions are invited, a fact-based synthesis statement explaining their decision and outlining what is needed to prepare a revision will be listed below. The following reviewer(s) agreed to reveal their identity: Kevin Foskett. Note: If this manuscript was transferred from JNeurosci and a decision was made to accept the manuscript without peer review, a brief statement to this effect will instead be what is listed below.

The authors have addressed all the issues raised by the Reviewers.
